# Long QT Syndrome and WPW Syndrome: A Very Rare Association between Two Causes of Sudden Cardiac Death in a Young Patient

**DOI:** 10.3390/jcm13030804

**Published:** 2024-01-30

**Authors:** Radu Gabriel Vătășescu, Silvia Deaconu, Corneliu Nicolae Iorgulescu, Gabriela Marascu, Bogdan Oprita, Alexandru Deaconu

**Affiliations:** 1 Faculty of Medicine, Carol Davila University of Medicine and Pharmacy, 050474 Bucharest, Romania; alexandru.deaconu@umfcd.ro; 2 Cardiology Department, Clinical Emergency Hospital, 014461 Bucharest, Romania; iorgulescu_corneliu@yahoo.com (C.N.I.); gabriela.marascu@yahoo.com (G.M.); 3 ARES Centers, 021967 Bucharest, Romania; si.deaconu@gmail.com; 4 Emergency Department, Clinical Emergency Hospital, 014461 Bucharest, Romania; bogdan.oprita@umfcd.ro

**Keywords:** WPW syndrome, long QT syndrome, sudden cardiac death

## Abstract

Long QT syndrome (LQT) and WPW syndrome are causes of sudden cardiac death (SCD) in the young, and their association has been rarely reported. A 26-year-old woman presented with recurrent syncope. Her ECG showed a short PR interval, wide QRS (150 ms) due to a delta wave, and QT prolongation (QT 580 ms, QTc 648 ms). ECG monitoring documented recurrent salvos of a self-terminating wide QRS tachycardia, generally slightly polymorphic, sometimes with “torsade des pointes” (TdP) appearance, which were linked to the syncopal/presyncope episodes. Electrophysiologic monitoring diagnosed a right para-hisian accessory pathway with a very short ERP (240 ms baseline, <200 ms after isoproterenol). The pathway was ablated successfully. Despite QRS narrowing (80 ms), QT prolongation persisted after ablation (QT 620 ms, QTc 654 ms), with short runs of TdP, despite beta-blocker treatment, which was increased to the maximal dosage. A dual-chamber implantable cardioverter defibrillator (ICD) was implanted. To our knowledge, this is the first case report of an association between LQT and WPW syndrome in which both conditions are associated with an increased risk of SCD.

## 1. Introduction

Long QT syndrome (LQT) and Wolff–Parkinson–White (WPW) syndrome are causes of sudden cardiac death (SCD) in young patients, and their association has been rarely reported. LQT is linked to 17 genes, but the majority of patients have a mutation in only three genes: KCNQ1 (LQT1) and KCNH2 (LQT2), which cause a loss of function, and SCN5A (LQT3), which causes a gain of function [1]. Type 1 long QT syndrome (LQT 1) is caused by a mutation of the KCNQ1 gene, and it is the most prevalent form of congenital LQT. This leads to a decrease in the function of the slowly activating delayed rectifier potassium channel (IKs) [2].

Syncope, which refers to a temporary loss of consciousness, is a clinical sign associated with an increased risk of SCD in various conditions related to structural or inherited heart disease [3]. Several primary electrical diseases, such as the WPW syndrome, the LQT syndrome, and the Brugada syndrome, can cause ventricular arrhythmia and may lead to syncope or SCD [3]. LQT is a condition with a repolarization abnormality, while WPW syndrome is associated with a depolarization abnormality. T-wave alternans (TWA) may be related to QT prolongation or LQT syndrome, and can also develop after catheter ablation of an accessory pathway.

High heart rates are believed to be connected to syncope and sudden death in LQT1 and tend to occur more frequently during physical activity or emotional situations [2]. All patients diagnosed with LQT are advised to take beta-blockers, ideally non-selective agents (nadolol or propranolol) [1]. These drugs have varying effects on the duration of the QT interval, which depends on the heart rate of the patient [2]. Patients who have suffered a cardiac arrest have a high risk of recurrence, even on beta-blocker therapy, supporting the use of an implanted cardioverter defibrillator (ICD) in such cases [1]. We report a rare case with both WPW and LQT syndromes.

## 2. Case Presentation

**Figure 1:**
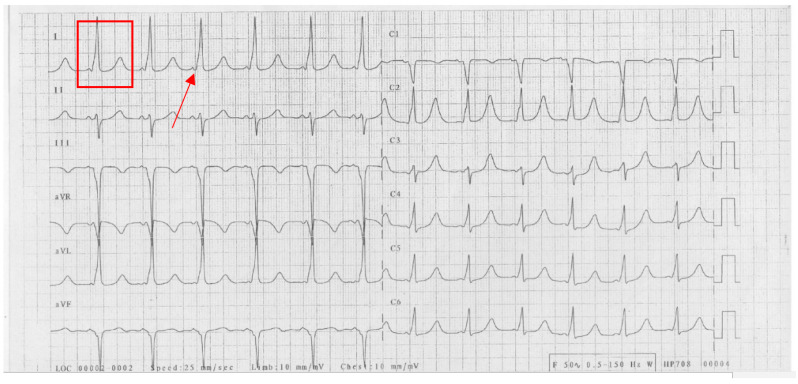
Patient’s baseline ECG. Red rectangle denotes the long QT interval; the arrow indicates the delta wave.

A 26-year-old woman presented with recurrent syncope. Clinical examination and echocardiography showed no signs of structural heart disease. The ECG in sinus rhythm showed a short PR interval, wide QRS (150 ms) due to a delta wave, and a QT interval increase (QT 580 ms, QTc 648 ms) (Figure 1).

Family history was consistent with relevant events: the patient had a sister who suddenly died at 18 years old while dancing, and her mother suffered from recurrent effort-induced syncope until 49 years old when she had menopause. Her mother’s ECG showed no pre-excitation with a long QT interval (QT 480 ms, QTc 550 ms). The patient’s syncope occurred occasionally without stress. According to the current guidelines of the European Society of Cardiology for the diagnosis of LQT syndrome [1], our patient meets the criteria for LQT syndrome (Table 1).

**Figure 2:**
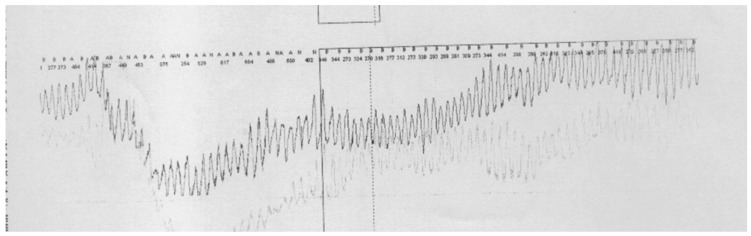
ECG monitoring revealing a wide QRS tachycardia episode, with a typical TdP appearance.

ECG monitoring documented several episodes of non-sustained wide QRS tachycardia. The arrhythmia was most frequently marginally polymorphic, occasionally with “torsade des pointes” (TdP) appearance, and it was associated with syncope/presyncope episodes (Figure 2).

Episodes of atrial flutter/fibrillation with rapid anterograde conduction over the accessory pathway were suspected and she was referred for electrophysiologic testing (EPS) and catheter ablation.

**Figure 3:**
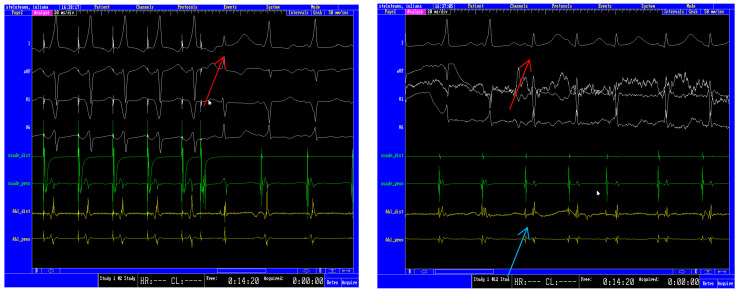
Electrophysiologic testing (cuad—tetrapolar catheter inside the proximal coronary sinus, Abl—tetrapolar ablation catheter). (**Left**): Programmed atrial stimulation revealing a right para-hisian accessory pathway with short AERP: 240 ms baseline: red arrow indicates a narrow QRS complex after a coupling interval of 240 ms; please note the discrete His potential on the ablation catheter unmasked by the loss of pre-excitation. (**Right**): RF current application leading to the disappearance of the delta wave. The red arrow denotes the first narrow QRS complex. The blue arrow shows the separation of the atrial and ventricular signals on the ablation catheter, proving the disappearance of the accessory pathway.

Electrophysiologic testing revealed a right para-hisian accessory pathway with a short anterograde effective refractory period (AERP) (240 ms baseline), which was successfully treated by RF ablation (Figure 3).

**Figure 4:**
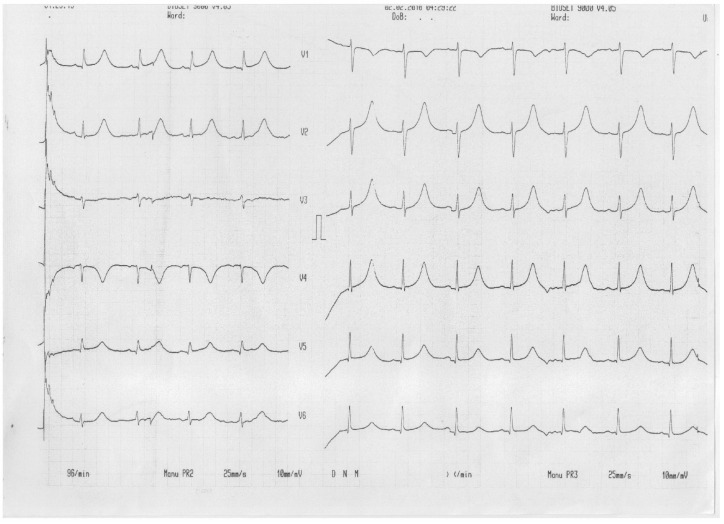
ECG after ablation and after maximal increase in the beta-blocker dose.

QT prolongation persisted after ablation (QT 620 ms, QTc 654 ms) without being affected by the QRS narrowing (80 ms), even after the initiation of treatment with propranolol (Figure 4).

Short runs of TdP were revealed by Holter monitoring, even after high-dose beta-blocker treatment (120 mg propranolol daily), resulting in a significant reduction of the resting heart rate. Genetic testing documented mutations in the KCNQ1 (KvLQT1) gene (502G > A i.e., Gly168Arg), thus diagnosing LQT1 syndrome.

The patient underwent a dual-chamber ICD implantation and was free of arrhythmia during the one-year follow-up, under beta-blocker therapy.

## 3. Discussion

This is a rare case with an association between WPW and LQT syndrome. The reported incidences of these syndromes in a general population are 1–3/100 for WPW [4,5] and 1/10,000 for LQT [6,7,8], so the coexistence of both syndromes is very rare. To our knowledge, only a handful of similar cases were reported [9,10,11]. However, those cases differed from ours in some aspects. Firstly, in our case, the accessory pathway had a short refractory period and was consequently associated with a risk of SCD, since the initiation of atrial fibrillation may have led to the induction of ventricular fibrillation due to the high ventricular rate being secondary to rapid conduction through the bypass tract. The documented arrhythmias associated with syncopal episodes—episodes of rapid, relatively regulated wide QRS tachycardia—may have also been caused by the accessory pathway, which was not the situation in the other two cases. Secondly, a familial history of LQT and SCD was only found in our case.

Two clinical forms of LQTS have been described: Romano–Ward syndrome (RWS), which follows an autosomal dominant pattern of inheritance, and Jervell and Lange-Nielsen syndrome (JLNS), which follows an autosomal recessive pattern of inheritance and is associated with sensorineural deafness (a recessive Romano–Ward syndrome associated with compound heterozygosity for two mutations in the KVLQT1 gene, as described by Lars Allan Larsen). LQT1 is the most common subtype of Romano–Ward syndrome, responsible for 30 to 35% of all cases. RWS can be differentiated from other forms of LQT syndrome by RWS’ sole involvement of the heart [12]. Given that the patient’s mother was not genetically tested to establish the diagnosis of long QT syndrome (she was only known to have a long QT interval), the patient’s sister died suddenly at a young age during physical activity, and the patient did not have hearing loss, we can only assume that our patient may have Romano Ward syndrome.

The overlap between WPW and LQT syndrome exponentially increases the global arrhythmic risk for our patient through multiple mechanisms. From one perspective, the accessory pathway can facilitate the appearance of TdP by increasing repolarization dispersion and further lengthening the QTc interval [13]. Previous studies have documented abnormal repolarization in patients with WPW syndrome, resulting in altered epicardial apicobasal activation-recovery intervals [14]. Furthermore, the altered activation sequence of the preexcitation rhythm is associated with a high dispersion of repolarization, which disappears after 1 month after successful conversion to a normal sinus rhythm [14].

Another possible mechanism that can facilitate the induction of ventricular arrhythmia in LQT syndrome is the increase in sympathetic tone induced by accessory pathway-mediated tachycardias. In turn, the augmented sympathetic tone decreases the refractory interval of atrial and ventricular tissue [15]. Moreover, sudden adrenergic stress increases the severity of arrhythmias in LQT syndrome [16]. This concept is supported by the observation that the beta-adrenoceptor blockade is reported to be the most effective in LQTS type 1 (as in the case of our patient) and only moderately effective in LQTS type 2 and LQTS type 3 [17].

The manifestation of LQT in patients is gene-specific, and those with LQT 1 usually experience cardiac events at a younger age. In fact, about 86% of LQT 1 patients have their first episode around the age of 20 [17]. Cardiac arrhythmias are often triggered by exercise or emotions in LQT 1 syndrome. This is due to the functions of IKs channels, which are activated by fast heart rates and catecholamines [16]. Patients with LQT 1 syndrome and the “loss-of-function type mutation” of IKs channels are expected to shorten their QT intervals less at fast heart rates. This can lead to early afterdepolarizations, which may then result in TdP via reentry [16].

Conversely, LQT syndrome increases the SCD risk mediated by the accessory pathway since it is associated with atrial fibrillation (AF) [18].

Although IKs play a crucial part in the repolarization of the heart, the medications currently utilized to treat LQT do not directly target it [12]. LQT 1 patients exhibit higher sensitivity towards stress- and exercise-induced arrhythmia compared to other subtypes of LQT [12]. Beta-blockers have proven to be notably effective for such patients [1,12]. During sympathetic activation in LQT 1, action potential prolongation is believed to occur due to inadequate upregulation of IKs in response to adrenergic stimulation, which is insufficient to offset the simultaneous increase in inward calcium current [12]. Adrenergic receptors are antagonized by beta-blockers, resulting in a reduction in the imbalance between potassium and calcium currents. This reduction, in turn, leads to a decreased likelihood of experiencing life-threatening arrhythmias [12].

The effects of beta-blockers on the QT and QTc intervals in LQT 1 are dependent on heart rate [2]. After beta-blockade, QT and QTc intervals were observed to shorten during faster heart rates induced by exercise. Similarly, beta-blockade led to an increase in these intervals during slower heart rates [2]. Equivalent protection in the LQT syndrome is not provided by all beta-blockers [19]. Extensive research has been conducted on propranolol, which is the standard beta-blocking agent. The results of these studies indicate that propranolol can either prevent or decrease lengthening in the transmural dispersion of repolarization when strong sympathetic stimulation occurs [19]. A multicenter study conducted by Chockalingam et al., which included patients with LQT syndrome who received beta-blocker therapy, has shown that propranolol and nadolol are significantly more effective than metoprolol in preventing cardiac adverse events in symptomatic patients. Propranolol was found to be more effective than both nadolol and metoprolol in reducing the cardiac repolarization time, especially in patients at high risk with significantly prolonged QTc intervals [19].

Albeit an exceptional association, it may be possible that in the future a genetic connection might be found between the embryologic mutation, which leads to accessory pathway formation and the simultaneous occurrence of repolarization abnormalities due to an ion channelopathy that can result in QT interval widening and TdP [9].

Radiofrequency ablation is curative for WPW while implantable cardioverter defibrillator (ICD) implantation is recommended for LQT associated with recurrent syncope, ventricular arrhythmia, sudden cardiac arrest, or a strong familial history of SCD. Furthermore, ICD implantation is indicated when a patient presents with sudden cardiac arrest or continues to experience syncope and/or ventricular arrhythmia, despite optimal pharmacological therapy, as syncopal events are associated with an increased risk of cardiac arrest [1]. Interestingly, our patient’s syncopal episodes occurred without stress, suggesting that arrhythmia associated with WPW syndrome might also be involved. Moreover, the episodes of wide QRS tachycardia that were recorded through Holter ECG monitoring were monomorphic as well as polymorphic, possibly suggesting two different mechanisms: antidromic AVRT and TdP in the LQT setting.

Although a few short episodes of TdP still occurred in the setting of persistent LQT under maximal beta-blocker therapy, our patient was symptom-free. This finding suggests a significant accessory pathway contribution to sustained wide-QRS arrhythmia, either in the form of rare antidromic tachycardia or symptomatic sustained orthodromic tachycardia leading to the induction of TdP in the presence of the LQT syndrome. However, since the patient had not been taking a beta-blocker prior to catheter ablation, it is unclear whether the ablation or addition of a beta-blocker was more effective in suppressing syncope. However, since she continued to have short runs of TdP despite a maximum beta-blocker dose, there is a strong indication that depolarization/repolarization anomalies associated with overt pre-excitation significantly contributed to her symptoms. The prediction of a 5-year absolute risk of the first life-threatening arrhythmic event (defined as aborted cardiac arrest, SCD, or appropriate ICD shock) using the 1-2-3-LQTS-Risk score was calculated at 0.82% [20]. Due to the presence of short runs of TdP after the ablation and under maximal beta-blocker treatment, we discussed further available therapeutic options with the patient. Since a wearable cardioverter defibrillator is not available in our country and is only a short-to-medium-term solution, and considering her family history, the patient opted for transvenous ICD implantation.

## 4. Conclusions

To our knowledge, this is the first case report of an association between LQT and WPW syndrome in which both conditions present a high risk of SCD. The association between the two rare diseases, one causing a depolarization abnormality and the other a repolarization abnormality, exponentially increases the global arrhythmic risk of our patient.

## Figures and Tables

**Table 1:** Modified long QT syndrome diagnostic score—adapted from the 2022 European Society of Cardiology guidelines for the management of patients with ventricular arrhythmias and the prevention of sudden cardiac death [1].

**ECG finding**	
**QTc ≥ 480 ms**	3.5
**= 460–479 ms**	2
**= 450–459 ms (in males)**	1
**≥ 480 ms during 4th minute of recovery from exercise stress test**	1
**Torsade de pointes**	2
**T-wave alternans**	1
**Notched T-wave in 3 leads**	1
**Low heart rate for age**	0.5
**Clinical history**	
**Syncope**	
**with stress**	2
**without stress**	1
**Family history**	
**Family member(s) with definite LQTS**	1
**Unexplained SCD at age < 30 years in first-degree family**	0.5
**Genetic finding**	
**Pathogenic mutation**	3.5

ECG—electrocardiogram, QTc—corrected QT interval (calculated by Bazett’s formula), LQTS—long QT syndrome, SCD—sudden cardiac death; red rectangles denote the criteria met by our patient; Our patient’s score is 7 points.

## Data Availability

Data are available upon request due to restrictions e.g., privacy or ethical restrictions. The data presented in this study are available upon request from the corresponding author. The data are not publicly available due to the General Data Protection Regulation.
